# Supplementary figures and images for: Identification of novel biomarkers in obstructive sleep apnea via integrated bioinformatics analysis and experimental validation
Source: PeerJ. 2023 Dec 4;11:e16608. doi: 10.7717/peerj.16608 (PMC10702330; doi:10.7717/peerj.16608)

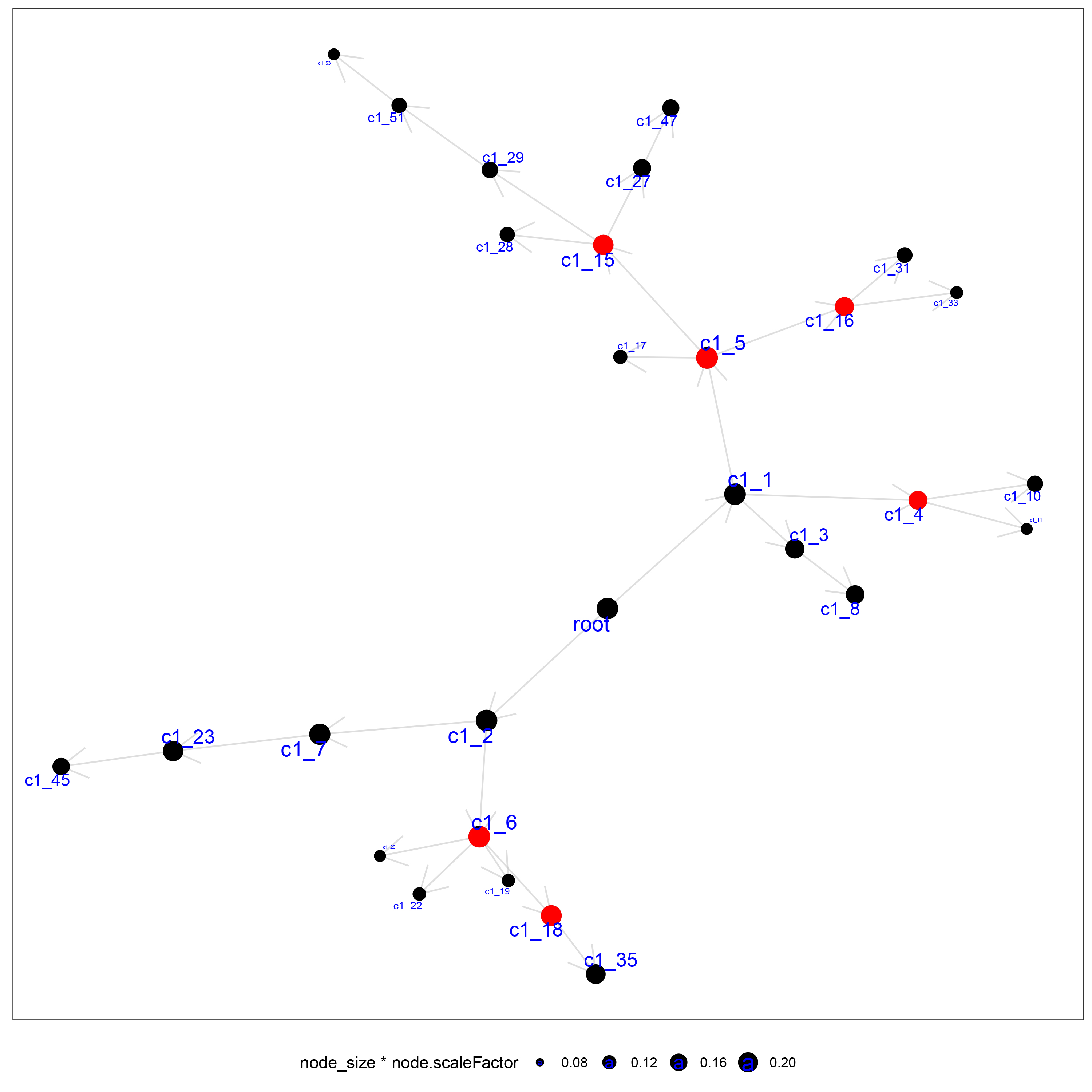

Supplement: Supplemental Information 3 — MEGENA co-expression network of differentially expressed genes, with larger nodes representing higher numbers of genes. [file peerj-11-16608-s003.jpg]

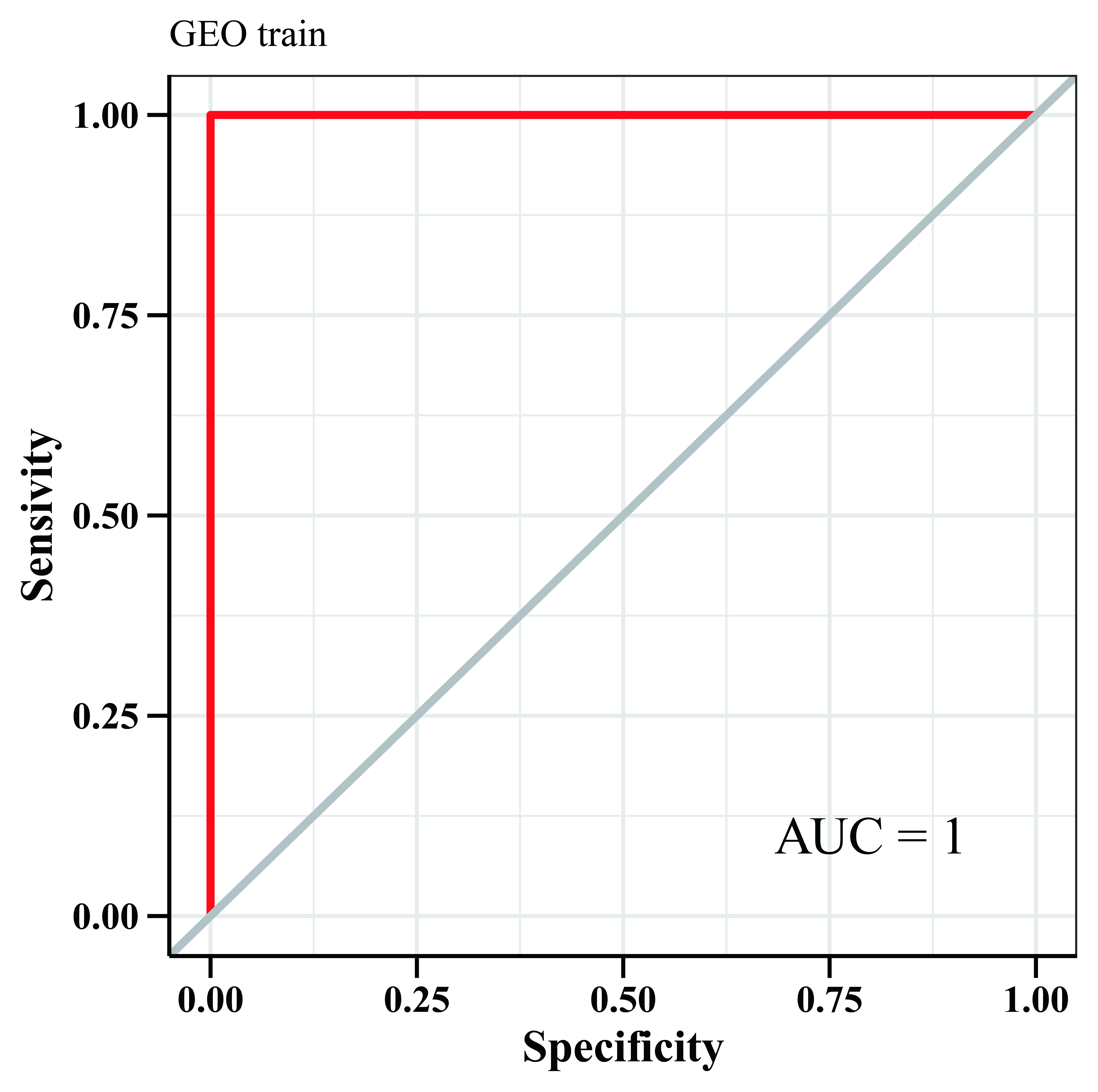

Supplement: Supplemental Information 4 — The predictive performance on the training set. [file peerj-11-16608-s004.jpg]
