# Supplementary material for: Identification of novel biomarkers in obstructive sleep apnea via integrated bioinformatics analysis and experimental validation
Source: PeerJ. 2023 Dec 4;11:e16608. doi: 10.7717/peerj.16608 (PMC10702330; doi:10.7717/peerj.16608)
Supplement: Supplemental Information 5 [file peerj-11-16608-s005.docx]

| Gene | Nucleotide Sequence (5′–3′) |
| --- | --- |
| C12orf54-F | AGCAGCAGAGAAGCACATCC |
| C12orf54-R | CATTAGCACCTGCTCCCACA |
| FOS-F | TCTCTAGTGCCAACTTTATCCC |
| FOS-R | GAGATAGCTGCTCTACTTTGCC |
| GPR1-F | GGAGCTCAGCATTCATCACA |
| GPR1-R | GACAGGCTCTTGGTTTCAGC |
| OR9A4-F | GCTCTGTAAACCTACGCCACA |
| OR9A4-R | ACAATGATGACCGTGTTTCCC |
| MYO5B-F | AACCAAGACACGTCAGAACGA |
| MYO5B-R | ACCCACAAAGGCAATTTACGTC |
| RAB39B-F | ATCGAGCCAGGAAAACGCAT |
| RAB39B-R | GTAGTAGGCGCGAGTGATGG |
| KLHL4-F | TGCTGAATGATCCGTCCCT |
| KLHL4-R | ACTCTAGCAAGGTCTTATACGTG |
| GAPDH-F | AATGGTGAAGGTCGGTGTGA |
| GAPDH-R | TGAGTGGAGTCATACTGGAACA |

Supplementary Table S1.Primer sequences in this study
