# Supplementary material for: Identification of novel biomarkers in obstructive sleep apnea via integrated bioinformatics analysis and experimental validation
Source: PeerJ. 2023 Dec 4;11:e16608. doi: 10.7717/peerj.16608 (PMC10702330; doi:10.7717/peerj.16608)
Supplement: Supplemental Information 6 [file peerj-11-16608-s006.docx]

Supplementary Table S2. Clinical characteristics of the patient

|  | Normal(n=8) | OSA(n=24) | t/x^2^/Z | p |
| --- | --- | --- | --- | --- |
| Age | 54.5±11.58 | 49.4±10.86 | 1.129 | 0.268 |
| Sex(%) |  |  | 2.709 | 0.100 |
| Male | 1(12.5) | 13(54.2) |  |  |
| Female | 7(87.5) | 11(45.8) |  |  |
| BMI(Min, Max) | 34.4(26.7,42.5) | 40.0(29.3,62.5) | -1.828 | 0.068 |
