# Supplementary material for: Identification of novel biomarkers in obstructive sleep apnea via integrated bioinformatics analysis and experimental validation
Source: PeerJ. 2023 Dec 4;11:e16608. doi: 10.7717/peerj.16608 (PMC10702330; doi:10.7717/peerj.16608)
Supplement: Supplemental Information 7 [file peerj-11-16608-s007.docx]

Supplementary Table 3. 29 characterized genes from MEGENA analysis

|  | gene |
| --- | --- |
| 1 | BMP7 |
| 2 | BTF3L4 |
| 3 | C12orf54 |
| 4 | CLDN1 |
| 5 | CRIPT |
| 6 | DSC3 |
| 7 | EGR1 |
| 8 | FOS |
| 9 | FOSB |
| 10 | GLB1 |
| 11 | GPR1 |
| 12 | IFI30 |
| 13 | KLHL4 |
| 14 | LRRC7 |
| 15 | MYO5B |
| 16 | NFASC |
| 17 | NFKBIZ |
| 18 | NIPSNAP3A |
| 19 | OR9A4 |
| 20 | PKHD1L1 |
| 21 | PLRG1 |
| 22 | PRCP |
| 23 | PTGS2 |
| 24 | RAB39B |
| 25 | RAP1B |
| 26 | RNF14 |
| 27 | SLC35A1 |
| 28 | TMEM106B |
| 29 | UCHL5 |
