# Supplementary material for: Identification of novel biomarkers in obstructive sleep apnea via integrated bioinformatics analysis and experimental validation
Source: PeerJ. 2023 Dec 4;11:e16608. doi: 10.7717/peerj.16608 (PMC10702330; doi:10.7717/peerj.16608)
Supplement: Supplemental Information 8 [file peerj-11-16608-s008.docx]

Supplementary Table 4. RiskScore coefficients and formulas for each gene

| Gene | coefficients |
| --- | --- |
| C12orf54 | -0.166676163325029 |
| FOS | -0.092114355266153 |
| GPR1 | 0.0569097301307626 |
| OR9A4 | -0.092114355266153 |
| MYO5B | -0.0494730342173479 |
| RAB39B | -0.0282427058551795 |
| KLHL4 | -0.00563988911553265 |

RiskScore= C12orf54 × (-0.166676163325029)+ FOS × (-0.092114355266153) + GPR1 × (-0.0569097301307626)+OR9A4 × (-0.092114355266153)+MYO5B × (-0.0494730342173479)+RAB39B × (-0.0282427058551795)+KLHL4 × (-0.00563988911553265)
